# Effect of Solidification Behavior on Microstructures and Mechanical Properties of Ni-Cr-Fe Superalloy Investment Casting

**DOI:** 10.3390/ma10030250

**Published:** 2017-03-01

**Authors:** Maodong Kang, Jun Wang, Haiyan Gao, Yanfeng Han, Guoxiang Wang, Shuxian He

**Affiliations:** 1 Shanghai Key Lab of Advanced High-Temperature Materials and Precision Forming, Shanghai Jiao Tong University, Shanghai 200240, China; kangmd518@sjtu.edu.cn (M.K.); gaohaiyan@sjtu.edu.cn (H.G.); yfhan@sjtu.edu.cn (Y.H.); gxwang@sjtu.edu.cn (G.W.); heshuxian@sjtu.edu.cn (S.H.); 2 State Key Laboratory of Metal Matrix Composites, Shanghai Jiao Tong University, Shanghai 200240, China; 3 Collaborative Innovation Center for Advanced Ship and Deep-Sea Exploration, Shanghai 200240, China

**Keywords:** solidification behavior, Ni-Cr-Fe superalloy, microstructures, mechanical properties

## Abstract

The effect of solidification behavior on the microstructures and mechanical properties of Ni-Cr-Fe superalloy investment casting is given. Metallographic and image analysis have been used to quantitatively examine the microstructures’ evolution. For the parts with the thickness of 3 mm and 24 mm, the volume fraction and maximum equivalent radius of the Laves phase increases from 0.3% to 1.2%, from 11.7 μm to 23.4 μm, respectively. Meanwhile, the volume fraction and maximum equivalent radius of carbides increase from 0.3% to 0.5%, from 8.1 μm to 9.9 μm, respectively. In addition, the volume fraction of microporosity increases from 0.3% to 2.7%. As a result, the ultimate tensile strength is reduced from 1125.5 MPa to 820.9 MPa, the elongation from 13.3% to 7.7%, and the quality index from 1294.2 MPa to 954.0 MPa, respectively. A typical brittle fracture is observed on the tensile fracture. As the cooling rate decreases, the microstructures become coarser.

## 1. Introduction

Ni-Cr-Fe superalloy is extensively used in aerospace engines, heavy-duty gas turbines, nuclear power plants, and petrochemical engineering owing to its microstructural stability, good oxidation resistance, and high excellent mechanical properties [1,2]. Due to its difficulty in hot deformation, investment casting (lost wax casting) has become the only way to produce large-scale complex thin-walled components of modern high-strength Ni-Cr-Fe superalloys in the past few decades. Investment casting is a favorable molding process for high-end casting production because of its advantages in excellent surface quality and dimensional accuracy. With the development of aerospace and land-based engine industries, complex-shape superalloy castings are increasingly in demand [3]. However, the section dimension of the casting changes greatly from one part to another owning to the complex structure, which often results in different solidification behavior, multifarious microstructure, and mechanical properties.

The effect of solidification behavior on the microstructure and mechanical properties of superalloy casting has been presented in much of the literature concerning the elemental segregation, phase selection and defect formation [4,5,6]. For instance, the solidification process of IN718 superalloy was studied by Antonsson [7], who found that the distribution of Nb element had a strong impact on the solidification sequence and microstructures. Litao Chang et al. [6] investigated the solidification behavior of 720Li superalloy, and pointed out that the formation of γ/γ-eutectic was related with the solute redistribution and enrichment of Al and Ti elements in the residual liquid. Ercan et al. [8] reported that the final microstructures of superalloy mostly depended upon the cooling rate, and increasing cooling rate greatly improved the mechanical properties of the superalloy due to the reduction in the size of primary and secondary dendrites. The influence of solidification variables on porosity was also investigated by Whitesell [9], who pointed out that the size and total porosity level depended strongly upon the withdrawal velocity, and microporosity growth appeared to be limited by the secondary and tertiary dendrite arms.

However, direct experimental evidence is limited to confirm the difference of microstructure and mechanical properties at the different thickness locations of Ni-Cr-Fe superalloy investment casting. In the present work, a systematic study was carried out to clarify the effect of solidification behavior in different parts on the microstructure evolution and mechanical properties of Ni-Cr-Fe superalloy investment casting.

## 2. Materials and Methods

### 2.1. Materials

The Ni-Cr-Fe superalloy used in the present study was cut from a cylindrical bar (Φ100 mm) fabricated by Central Iron and Steel Research Institute, China, with the chemical composition listed in Table 1.

### 2.2. Investment Casting

**Figure 1:**
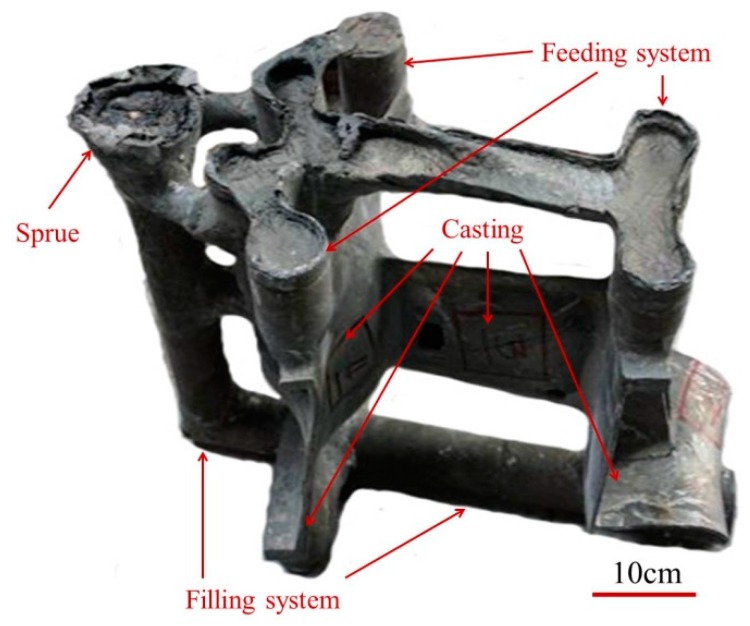
Photo of the superalloy casting.

In the present study, ceramic slurry and SiO_2_ sands were smeared successively to the surface of the designed wax, repeating ten times, and forming the disposable ceramic shell. The wax in the ceramic shell was removed at the pressure of 770 kPa by steam, leaving the hollow cavity inside the ceramic shell. Then, the ceramic shell was put into the rectangular sandbox and filled with SiO_2_ sands. A homemade middle frequency induction investment casting furnace equipped with a vacuum chamber was used for melting and casting, and the current frequency is 1000 Hz. The superalloy melt was refined at 1600 °C for 10 min, then waiting for pouring at 1500 °C The ceramic shells, together with the sandbox, were heated to 1000 °C and held for 20 h. Then, the superalloy melt was poured into the ceramic shell in seven seconds, then breaking the vacuum immediately. Ten hours later, the ceramic shell was destroyed to obtain the superalloy casting. Figure 1 shows the morphology of the superalloy casting with the pouring system.

### 2.3. Heat Treatment

After the filling and feeding system being cut, the superalloy casting was treated with standard heat treatment via homogenizing treatment at 1095 °C for 2 h, solution at 955 °C for 1 h and aging treatment consisting of 720 °C for 8 h/furnace cooling at 55 °C·h^−1^ to 620 °C for 8 h before air cooling to room temperature.

### 2.4. Microstructure Analysis

**Figure 2:**
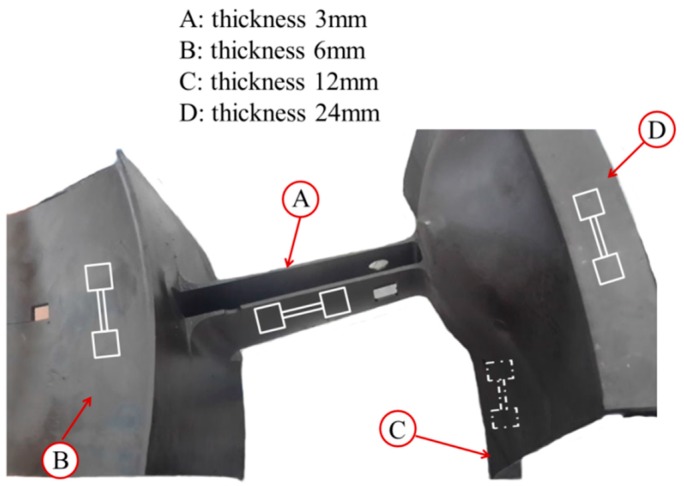
The sampled positions at the superalloy casting.

The specimens for microstructure observation and quantitative analysis were taken from the superalloy casting with different thickness (3, 6, 12, and 24 mm) on behalf of different solidification behaviors, as shown in Figure 2.

Standard metallographic procedures were carried out to prepare the specimens for microstructural characterization. Quantitative metallographic analysis was carried out using optical microscopy (OM; Zeiss AXiolam MRC5, arl Zeiss AG, Oberkochen, Germany) with DT2000 commercial image analysis system. The composition analysis of the precipitates were carried out using energy dispersive spectroscopy (JSM-7600, EDS, Japan Electronics Corp., Tokyo, Japan). The maximum value within an area of 1 mm × 1 mm at the magnification of 50 is defined as microporosity index according to the China National Standard (GB/T 14999.7-2010). Then, the metallographic specimens were chemically etched with a combination of CuSO_4_ (15 g), HCl (50 mL), and H_2_SO_4_ (3.5 mL) solution for 10 s. The details of the experimental procedure were reported elsewhere [10,11]. The quantitative analysis of the Laves phase and carbides were carried out at the magnification of 200× and 500×, respectively. The size of the Laves phase was calculated according to GB/T 14999.7-2010. At least six images (2584 × 1936 pixels) were measured in order to provide a suitable statistical analysis.

### 2.5. Properties Test

**Figure 3:**
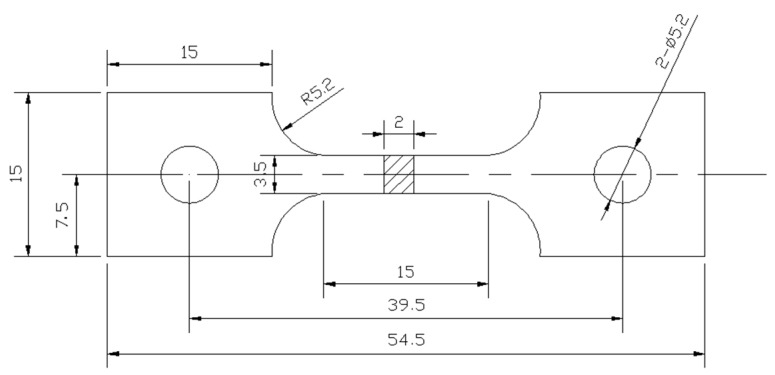
Schematic illustration of the tensile specimens (all of the dimensions are in mm).

Sheet specimens from the superalloy casting with different thickness as labelled in Figure 2 were machined according to Figure 3. It is worth mentioning that the surface fine grain zone of the superalloy casting was cut off to eliminate the skin effect. Then, tensile tests were carried out using a Shimatzu mechanical testing machine at a constant strain rate of 4.2 × 10^−4^ s^−1^ at room temperature in open air. Each test result reported in this study was the mean values obtained from at least three specimens. In addition, the fracture morphology of the failed tensile specimens was examined by scanning electronic microscope (SEM) to determine the failure mechanism.

## 3. Results and Discussion

In this section, experimental results and discussion are presented including the microstructures, Laves phase, carbides, microporosity, and tensile properties.

### 3.1. Microstructures of Superalloy

**Figure 4:**
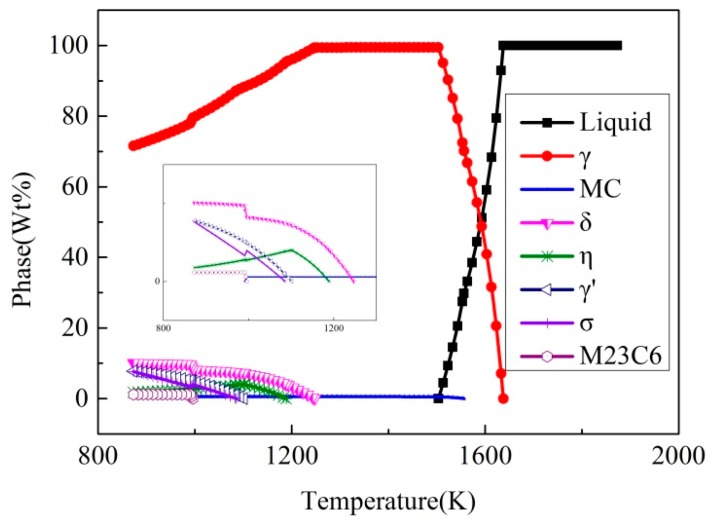
Phase fraction of the superalloy during equilibrium solidification.

Figure 4 shows the phase fraction of the superalloy under equilibrium solidification conditions calculated by JMatPro software (Sente Software Ltd., Guildford, UK). It is seen that there are so many phases in the alloy. However, investment casting is a non-equilibrium solidification process. Formation of non-equilibrium phases in the casting mainly depends on the local solidification behavior.

**Figure 5:**
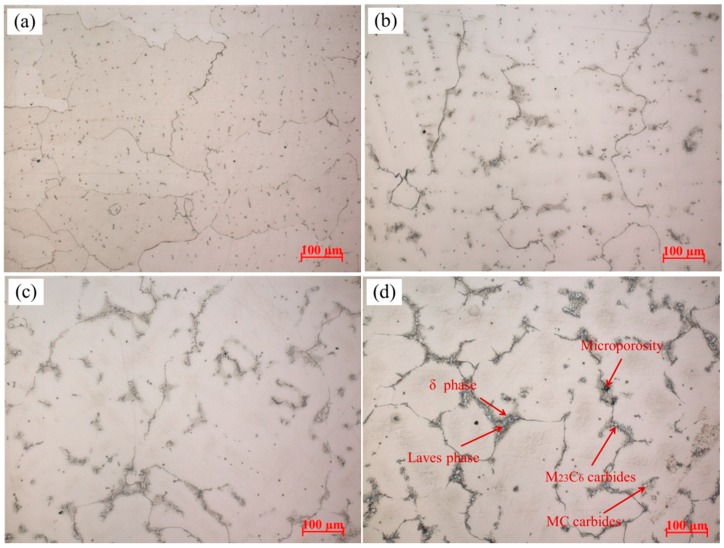
Optical microstructures of the superalloy casting (**a**) 3 mm; (**b**) 6 mm; (**c**) 12 mm; and (**d**) 24 mm.

**Figure 6:**
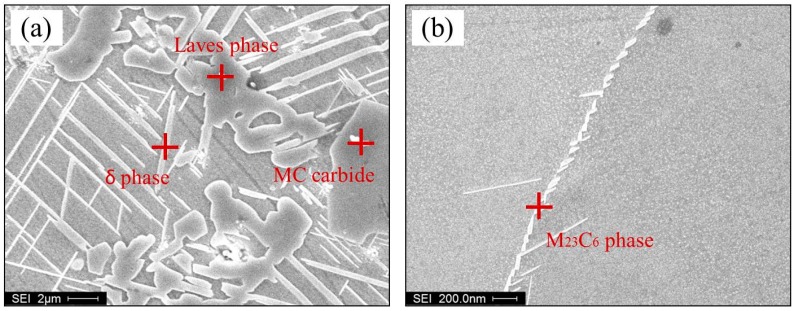

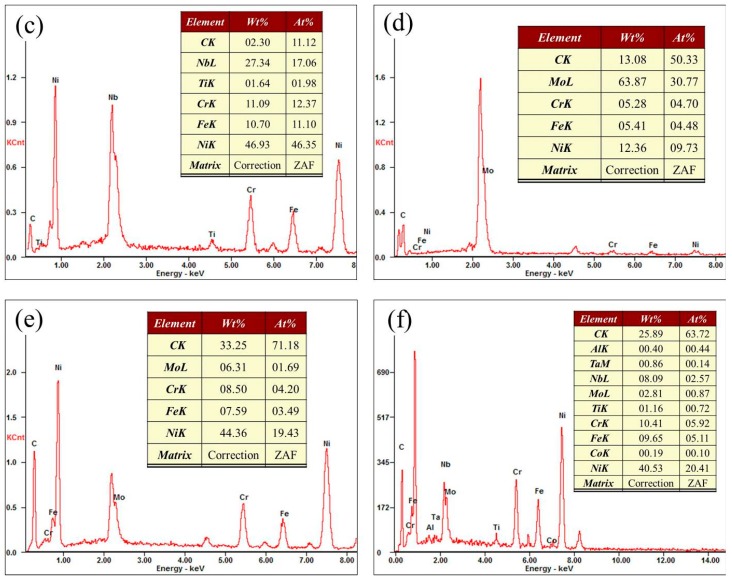
EDS analysis of precipitates. (**a**,**b**) Morphology of precipitates; (**c**) Laves phase; (**d**) MC carbides; (**e**) δ phase; and (**f**) M23C6 carbides.

Figure 5 illustrates the microstructures of different positions of the Ni-Cr-Fe superalloy casting as shown in Figure 2. The typical solidification microstructures of Ni-Cr-Fe superalloy are γ matrix, carbides, Laves phase, δ phase, and microporosity [12], and their EDS analysis is presented in Figure 6. As seen from Figure 5a, randomly distributed carbides present in interdendritic regions, and grain boundaries, and almost no Laves phase and microporosity appear. Along with the thickness increases, the Chinese script Laves phase is precipitated massively as distinct “islands” in interdendritic regions, and the needle-like δ phase grown along the surface of the Laves phase. Furthermore, the mass and size of these phase increase from 3 mm to 24 mm as shown in Figure 5a–d. Besides, large-scale microporosity also appeared in interdendritic regions, which is usually near the Laves phase as shown in Figure 5d. The observed different microstructures at different thickness result from different solidification behavior. The solidification behavior of the superalloy casting is related to solidification sequence, which is mainly decided by local cooling rate. The cooling rate usually decreases with increasing the wall thickness of the casting, which can be indirectly reflected by the solidification microstructure.

### 3.2. Characteristics of Laves Phases

In traditional investment casting, the Laves phase is an unavoidable terminal solidification phase in Ni-Cr-Fe superalloys. The Laves phase is defined as a brittle, topologically close-packed (TCP) structure that is easier to fracture than the matrix when the local stress is built up by an advancing crack [13]. The fractured Laves phase often creates a microcrack, which would decrease the mechanical properties significantly. Thus, the amount and size of the Laves phase plays a significant role in determining the mechanical properties of Ni-Cr-Fe superalloys.

**Figure 7:**
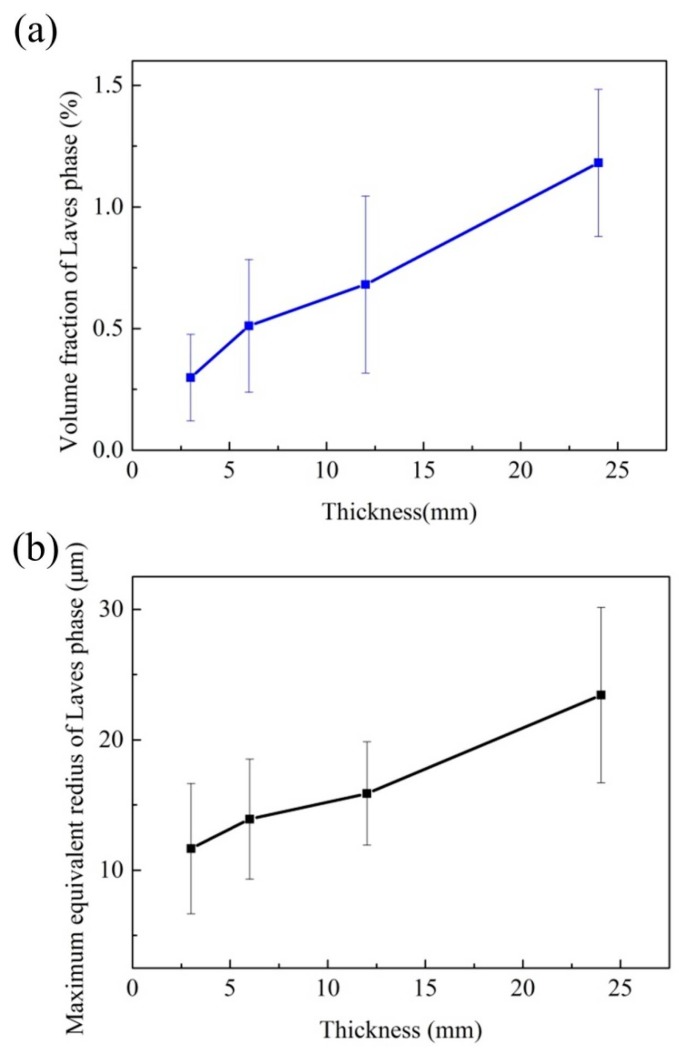
(**a**) The volume fraction and (**b**) the maximum equivalent radius of the Laves phase with different wall thicknesses.

Figure 7 summarizes the Laves phase of the different positions in the superalloy casting under the heat treatment conditions. The discussed characteristic parameters here are the volume fraction and the maximum equivalent radius of the Laves phase. The volume fraction of the Laves phase as plotted in Figure 7a, rising from 0.3% to 1.2%. It is reported that the cooling rate can strongly influence the extent of Nb segregation and, hence, the amount of the Laves phase [14]. In this case, the cooling rate decreases with the wall thickness increasing, and the distribution coefficient of Nb is less than unity, which would result in element Nb enrichment in the residual liquid. Consequently, the Laves phase is increased. The amount the Laves phase increases as the cooling rate decreases seems to be in agreement with Ling [15], who reported the relationship between the amount of the Laves phase and cooling rate experimentally and theoretically for Ni-Cr-Fe superalloys.

In this study, the equivalent radius is defined as a circle of equal area to the tortuous Laves phase, and the maximum equivalent radius of the Laves phase observed at the different positions of the superalloy casting is shown in Figure 7b. The maximum equivalent radius of the Laves phase was gradually increased from 11.7 μm to 23.4 μm. It is indicated that the maximum equivalent radius of the Laves phase also increases with a decreasing cooling rate. On one hand, the decreased cooling rate would increase the element segregation, as well as decrease constitutional undercooling; on the other hand, a thicker location can supply enough space for Laves phase growth, the two aspects work together to result in an emerging large-scale Laves phase. In addition, Radhakrishna pointed out that the morphologies of the Laves phase were also related to dendrite morphologies [16]. Large-scale Laves phase is accompanied with the coarser dendrites, which are often associated with serious microporosity, bulky carbides, and more needle-like δ phases. All of those metallurgical changes of the microstructures are responsible for poor mechanical performance.

### 3.3. Characteristics of Carbides

The characteristics of carbides contribute to the mechanical properties of the superalloy at room/elevated temperature, and their forms in the superalloy are classified as MC and M_23_C_6_ carbides. The “M” constitution in these carbides is mainly Nb with other elements, such as Ti and Mo elements [17]. Primary MC Carbides located inside the γ matrix and at the grain boundaries usually exhibit random, blocky, and script morphology [18], and M_23_C_6_ often precipitates in grain boundaries as shown in Figure 5.

**Figure 8:**
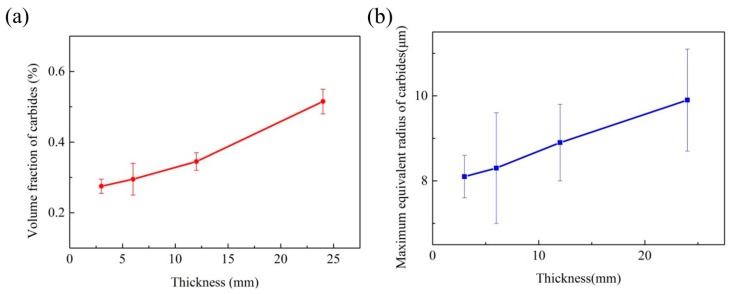
(**a**) The volume fraction and (**b**) the maximum equivalent radius of carbides with different wall thicknesses.

As shown in Figure 8a, the volume fraction of total carbides increases from 0.3% to 0.5%. In fact, the solute segregation, morphology of dendrite microstructure, together with local solidification time controls the carbides formation and growth. Solute segregation controls the carbide’s growth by providing carbide-forming elements. In detail, the microsegregation level has a close relation with the cooling rate; Liu also pointed out that the microsegregation level increases with the cooling rate increasing at lower cooling rates, which often result in larger-sized carbides [19]. The morphology of the γ matrix provides the interdendritic regions and grain boundaries for carbide growth. Moreover, the growth of carbides is a time-dependent process. In the present study, local solidification time increased along with an increase in the thickness. In summary, the solute supersaturating level of carbide-forming elements was improved, and the γ matrix also became coarser. Therefore, the increment of the volume fraction of carbides is quite expected.

The maximum equivalent radius of carbides is also useful in characterizing the structure changes of carbides. Solidification behavior can influence the maximum equivalent radius of carbides in this work, as shown in Figure 8b. It increased from 8.1 μm to 9.9 μm, with the thickness increasing. The existence of fine granular carbides in the matrix after standard heat treatment is helpful for the improvement of UTS and creep resistance by preventing the grain boundary from sliding (pinning effect) [20]. However, large-scale blocky carbides are the crack source of high temperature tension for Ni-Cr-Fe superalloys, because cracks may be initiated and extend easily at the interface between the carbides and the γ matrix [21].

### 3.4. Microporosity

**Figure 9:**
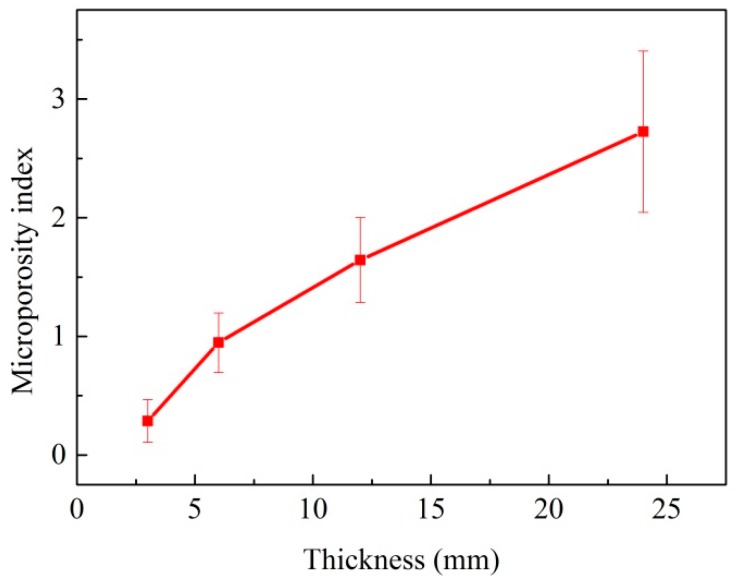
The volume fraction of microporosity as a function of the wall thickness.

In modern superalloys with low gas content, microporosity mostly forms due to solidification shrinkage and the amount of microporosity mainly depends on the feeding condition at the last stage of solidification. Figure 9 presents the microporosity index of the superalloy casting with different thicknesses. It can be concluded that the microporosity index gradually increased from 0.3 to 2.7. In this case, the results show that the microporosity index increase as the cooling rate decreased. Lower cooling rate increases the grain size and the solidification time of the superalloy casting. Firstly, coarser grain size could close more isolated liquid at the last stage of solidification, and provide large dendrite arm spacing, as well as intergranular regions for microporosity growth; secondly, more Al and Ti elements would enrich in interdendritic regions due to a longer solidification time because the distribution coefficients of them are all less than unity. However, Al, Ti, and Co could lead to increased microporosity in superalloys [22]. Thirdly, it is worth mentioning that 3-mm thin sections require little feeding, and the feeding can be compensated by surface depressions [23]. The three factors mentioned above could work together in the solidification process, and result in a microporosity index increase along with thickness increase.

**Figure 10:**
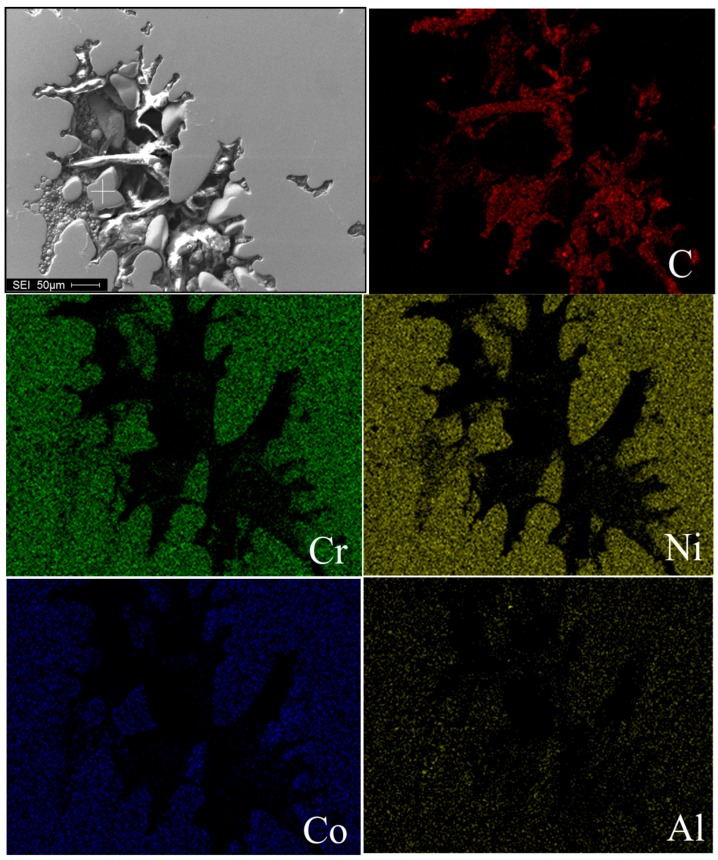

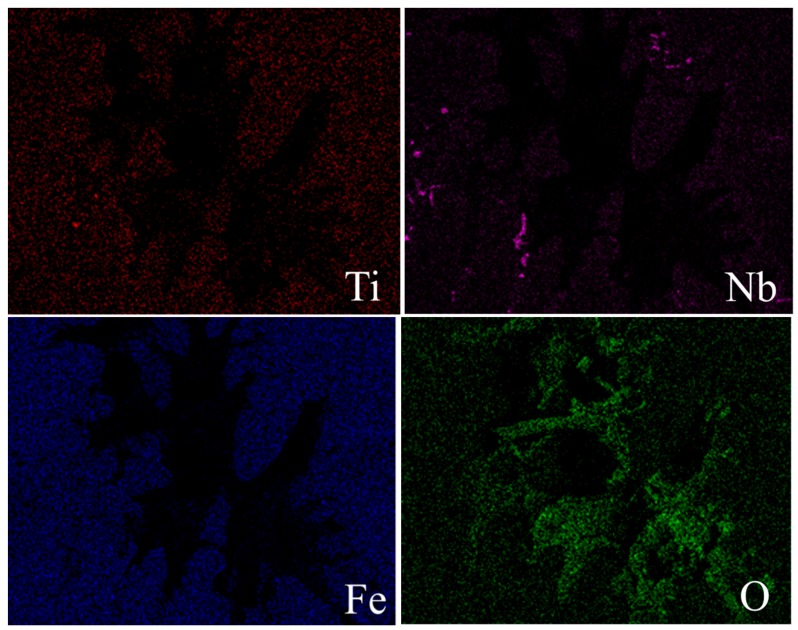
The elements mapping of the superalloy around a microporosity of one.

In the turbulence of the casting pouring process, Campbell pointed out that the oxide bifilms could be created, and some bifilms may decohere on one side to create microporosity [23]. To further identify the formation mechanism of microporosity in the superalloy casting, the main elements mapping of the superalloy and the inclusion of oxygen were performed as shown in Figure 10. It can be seen that a mass of oxygen emerge around the microporosity, which affirmed that the microporosity formation in the superalloy casting are the result of oxide bifilms and solidification shrinkage together.

### 3.5. Mechanical Properties

**Figure 11:**
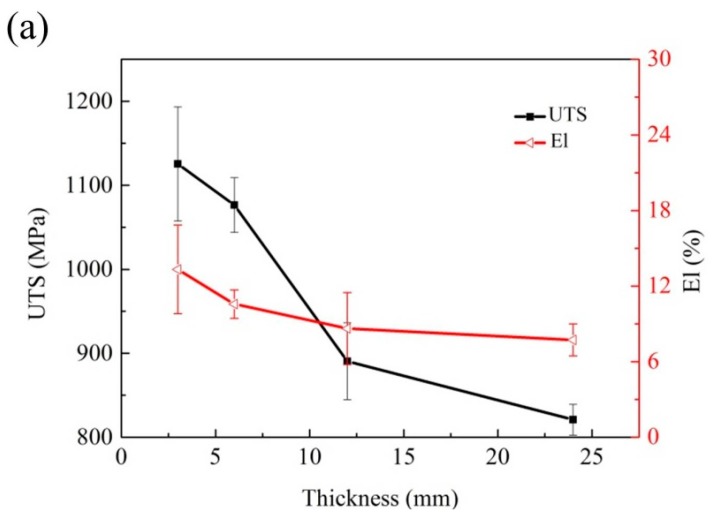

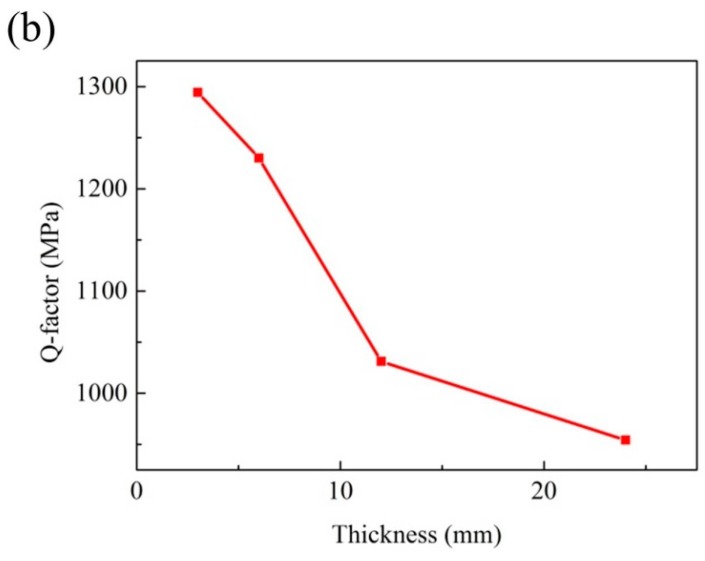
The tensile properties of the superalloy casting (**a**) tensile properties; and (**b**) Q-factor.

The tensile properties of Ni-Cr-Fe superalloy depend on many parameters, such as alloying elements, Laves phase, carbides, and microporosity. The results of the tensile properties at room temperature, obtained from different sections are shown in Figure 11a. It turned out that the solidification behavior influences the tensile properties significantly. With the decrease of the cooling rate, the ultimate tensile strength (UTS) and elongation (El) decrease. In more detail, the UTS decreased from 1125.5 MPa to 820.9 MPa, and the El decreased from 13.3% to 7.7%, respectively.

The quality index (Q) is taken to be more descriptive of the true tensile properties of casting than either the ultimate tensile strength (UTS) or the elongation (El) alone. Q is defined as a semi-logarithmic plot of UTS verse El to fracture and it is expressed as follows:

Q = UTS (MPa) + 150 × log (δ)
(1)

The quality index Q determined for each thickness specimens is showed in Figure 11b. For all conditions, increased Q indicates an improvement in the tensile properties. With the thickness increasing, the quality index of the superalloy casting decreases from 1294.2 MPa to 954.0 MPa. It is consistent with the observation that Q is sensitive to variations in the solidification behavior.

**Figure 12:**
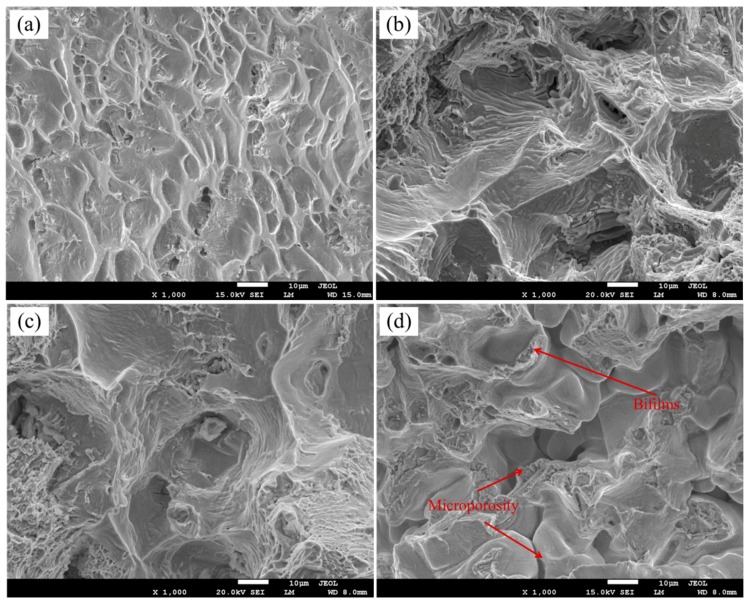
SEM micrographs showing the typical fracture on the tensile specimens (**a**) 3 mm; (**b**) 6 mm; (**c**) 12 mm; and (**d**) 24 mm.

To better understand the failure mechanism, a systematic fractographic analysis was performed by SEM on the fractured specimens. Figure 12 shows the fractographs of the four tensile specimens and the clearly different fracture surfaces depending on the solidification behavior. For the short solidification time specimen, a large amount of dimples present in the fracture surface as shown in Figure 12a, and this contribute to excellent tensile properties. It is obvious that the failure mode of the long solidification time specimen was microporosity-induced type as shown in Figure 12d, resulting in poor tensile properties of the specimens. However, the fracture surface is rough for the middle solidification time specimen as shown in Figure 12b,c. Similarly with Campbell’s research, bifilms were observed in fracture surface. The tensile failure of the specimen could take place along the unbonded bifilm interfaces. However, there are no obvious differences between 6 mm specimen and 12 mm specimens in fracture surface as shown in Figure 12b,c, and as the solidification time increases, the smooth flat areas become small and the fracture presents more casting defects, such as microporosity and bifilms in Figure 12d.

**Figure 13:**
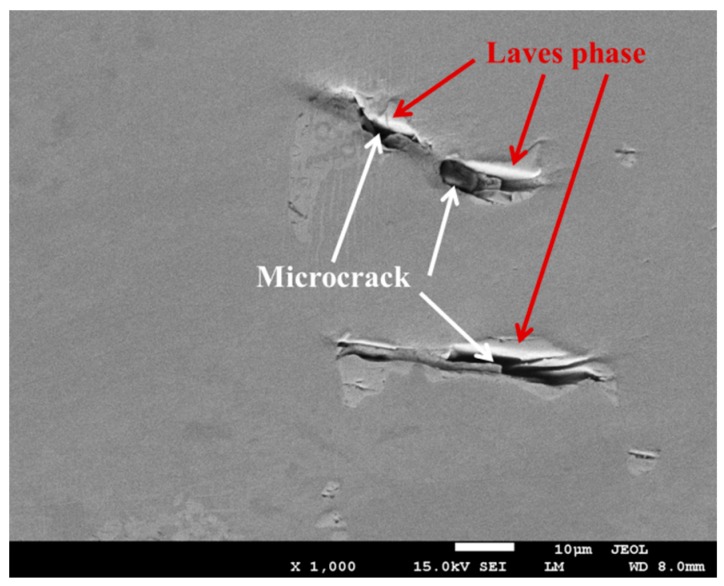
Back-scattered electron images of the longitudinal section from the specimen.

In the absence of microporosity specimens, the inherent brittle nature of the Laves phase leads to poor tensile ductility, fracture toughness, fatigue, and creep properties in the superalloy casting. The SEM examination on the longitudinal sections indicated that the crack propagated preferentially along the interdendritic region where the alignment of the Laves phase was present. Brittle microcracks that initiated from the Laves phase were observed, as shown in Figure 13.

## 4. Conclusions

The effect of solidification behavior on the microstructure and mechanical properties of Ni-Cr-Fe superalloy investment casting is investigated. The following conclusions can be drawn:

(1) Due to different solidification behavior, the microstructures and mechanical properties of the Ni-Cr-Fe superalloy investment casting greatly changes from 3 mm to 24 mm.
(2) As the thickness increases, the volume fraction of the Laves phase rises from 0.3% to 1.2%. The maximum equivalent radius of the Laves phase increases from 11.7 μm to 23.4 μm. The volume fraction and maximum equivalent radius of carbides increase from 0.3% to 0.5%, from 8.1 μm to 9.9 μm, respectively. In addition, the volume fraction of microporosity increases from 0.3% to 2.7%.
(3) The UTS reduced from 1125.5 MPa to 820.9 MPa, the El decreased from 13.3% to 7.7%, and the quality index (Q) reduced from 1294.2 MPa to 954.0 MPa. A typical brittle fracture mode was observed on the tensile fracture surface. As the solidification rate decreases, the smooth flat areas become small, and cracks propagated preferentially along the Laves phase in the absence of microporosity.

## Figures and Tables

**Table 1:** Composition of the experimental superalloy (mass percent, %).

C	Cr	Ni	Co	Mo	Al	Ti	Nb	Ta	Fe
0.06	19.43	52.09	0.18	3.15	0.41	1.06	4.36	0.08	Balance

## References

[1] Reed R.C. (2006). The Superalloys: Fundamentals and Applications.

[2] Cao W.D., Kennedy R.L. (2005). New developments in wrought 718-type superalloys. Acta Metallurg. Sin..

[3] Bayha T.D., Lu M., Kloske K.E. (2005). Investment Casting of Allvac 718 Plus Alloy. Superalloys 718, 625, 706 and Various Derivatives.

[4] Kavoosi V., Abbasi S.M., Mirsaed S.M.G., Mostafaei M. (2016). Influence of cooling rate on the solidification behavior and microstructure of IN738LC superalloy. J. Alloys Compd..

[5] Rahimian M., Milenkovic S., Sabirov I. (2013). Microstructure and hardness evolution in MAR-M247 Ni-based superalloy processed by controlled cooling and double heat treatment. J. Alloys Compd..

[6] Chang L., Jin H., Sun W. (2015). Solidification behavior of Ni-base superalloy Udimet 720Li. J. Alloys Compd..

[7] Antonsson T., Fredriksson H. (2005). The effect of cooling rate on the solidification of INCONEL 718. Metall. Mater. Trans. B.

[8] Karakose E., Keskin M. (2015). Effect of microstructural evolution and elevated temperature on the mechanical properties of Ni-Cr-Mo alloys. J. Alloys Compd..

[9] Whitesell H.S., Overfelt R.A. (2001). Influence of solidification variables on the microstructure, macro segregation, and porosity of directionally solidified Mar-M247. Mater. Sci. Eng. A.

[10] Chen Q., Chen Z., Liu F., Cui R.X., Liang T. (2015). The investigation of recrystallization developed in the largely undercooled Ni-3 at.% Sn alloy. J. Alloys Compd..

[11] Ye X., Hua X., Wang M., Lou S. (2015). Controlling hot cracking in Ni-based Inconel-718 superalloy cast sheets during tungsten inert gas welding. J. Mater. Proc. Technol..

[12] Kang M., Wang J., Wang K., Ling L., Sun B. (2014). Tensile properties and microstructures of investment complex shaped casting. Mater. Sci. Technol..

[13] Il Kwon S., Bae S.H., Do J.H., Jo C.Y., Hong H.U. (2016). Characterization of the Microstructures and the Cryogenic Mechanical Properties of Electron Beam Welded Inconel 718. Metall. Mater. Trans. A.

[14] Wang L., Li Y., Dong J., Zhang M. (2000). Effect of cooling rates on segregation and density variation in the mushy zone during solidification of superalloy inconel 718. Chem. Eng. Commun..

[15] Ling L.S.B., Han Y.F., Zhou W., Gao H.Y., Shu D., Wang J., Kang M.D., Sun B.D. (2015). Study of Microsegregation and Laves Phase in INCONEL718 Superalloy Regarding Cooling Rate during Solidification. Metall. Mater. Trans. A.

[16] Radhakrishna C.H., Prasad Rao K. (1997). The formation and control of Laves phase in superalloy 718 welds. J. Mater. Sci..

[17] Chang S.-H. (2009). In situ TEM observation of γ′, γ″ and δ precipitations on Inconel 718 superalloy through HIP treatment. J. Alloys Compd..

[18] Shahriari D., Sadeghi M.H., Akbarzadeh A. (2009). Precipitate Dissolution during Heat Treatment of Nimonic 115 Superalloy. Mater. Manuf. Process..

[19] Liu L., Sommer F., Fu H.Z. (1994). Effect of solidification conditions on MC carbides in a nickel-base superalloy IN 738 LC. Scr. Metall. Mater..

[20] Appa Rao G., Satyanarayana D.V.V. (2011). Influence of HIP processing on microstructure and mechanical properties of superalloy Udimet 720LI. Mater. Sci. Technol..

[21] Lu X., Du J., Deng Q. (2013). In situ observation of high temperature tensile deformation and low cycle fatigue response in a nickel-base superalloy. Mater. Sci. Eng. A.

[22] Lecomte-Beckers J. (1988). Study of microporosity formation in nickel-base superalloys. Metall. Mater. Trans. A.

[23] Campbell J. (2011). Complete Casting Handbook: Metal Casting Processes, Techniques and Design.

